# Prognostic value of combined preoperative lactate dehydrogenase and alkaline phosphatase levels in patients with resectable pancreatic ductal adenocarcinoma

**DOI:** 10.1097/MD.0000000000004065

**Published:** 2016-07-08

**Authors:** Fei Ji, Shun-Jun Fu, Zhi-Yong Guo, Hui Pang, Wei-Qiang Ju, Dong-Ping Wang, Yun-Peng Hua, Xiao-Shun He

**Affiliations:** a Organ Transplant Center, the First Affiliated Hospital, Sun Yat-sen University, Guangdong Provincial Key Laboratory of Organ Donation and Transplant Immunology, Guangdong Provincial international Cooperation Base of Science and Technology, Guangzhou, P.R. China; b Department of Medical Records; c Department of Liver Surgery, the First Affiliated Hospital, Sun Yat-sen University, Guangzhou, P.R. China.

**Keywords:** alkaline phosphatase, biomarkers, lactate dehydrogenase, pancreatic ductal adenocarcinoma, prognosis

## Abstract

Serum enzymes, including lactate dehydrogenase (LDH) and alkaline phosphatase (ALP), have recently been reported to play important roles in tumor growth. Increases in LDH and ALP have been confirmed to predict poor prognosis in patients with various cancers. However, their prognostic value in pancreatic cancer has not been well studied. Therefore, we reviewed the preoperative data on LDH and ALP in 185 pancreatic ductal adenocarcinoma (PDAC) patients who underwent surgery between July 2005 and December 2010 to explore the prognostic value of these markers. The cutoff points were determined based on the upper limit of their normal values. The Chi-square test was used to analyze the relationships between LDH/ALP and clinical characteristics. Univariate and multivariate analyses were performed to identify the predictive value of the above factors for disease-free survival (DFS) and overall survival (OS). We found that elevation of LDH was related to carbohydrate antigen 19-9 (CA19-9), lymph node involvement, tumor size, TNM, distant metastasis, and recurrence. Additionally, ALP was correlated to perineural invasion. After multivariate analysis, LDH and ALP were identified as independent prognostic factors for DFS and OS, and elevation of LDH/ALP was correlated with poor DFS and OS. Notably, there was a positive correlation between LDH and ALP. The predictive power of LDH combined with ALP was more sensitive than that of either one alone. Therefore, we conclude that the preoperative LDH and ALP values are prognostic factors for PADC, and the prognostic accuracy of testing can be enhanced by the combination of LDH and ALP

## 1 Introduction

Pancreatic cancer is the 5th most common cancer and the 4th leading cause of cancer-related mortality worldwide,^[1]^ with approximately 227,000 deaths per year.^[2]^ Sixty to seventy percent of newly diagnosed pancreatic cancer patients present with advanced disease. The 5-year survival among these patients is only 5% to 7%,^[3,4]^ and the vast majority of patients survive less than 1 to 2 years. Even following curative surgery, 90% of patients progress in 12 to 18 months.^[5–9]^ This poor outcome is often attributed to late stage presentation, which is due to the lack of clinically useful biomarkers that can detect pancreatic cancer in the early stages. It is therefore essential and urgent to identify risk factors in patients to provide better individual therapies and improve clinical outcomes.

The transformation of normal cells to cancer cells or the proliferation of cancer cells always leads to abnormal serum enzyme synthesis, sometimes even before the changes in tumor morphology, that is, before it is clinically detectable.^[10]^ Thus, more and more attention has been paid to serum enzymes. Lactate dehydrogenase (LDH) participates in the conversion of pyruvate to lactate, providing NAD^+^ for continued glycolysis.^[11]^ It is expressed in all tissues and contains 2 subunits, A and B. The 2 subunits combine to form 5 isoenzymes (LDH1 to LDH5) that are selectively distributed in the tissues and serum. Additionally, LDH is a marker of tumor burden as it is required for tumor maintenance. The link between LDH-A and the oncogene c-MYC has been confirmed, and elevated LDH levels have also been confirmed in many cancers, such as germ cell tumors, lymphoma, melanoma, and renal cell carcinoma.^[10,12–14]^ Alkaline phosphatase (ALP) is another hydrolase comprising several isoenzymes that catalyze the hydrolysis of phosphate esters in an alkaline environment, generating an organic radical and inorganic phosphate. ALP is localized to the liver, bone, intestines, placenta, and kidneys and is also found in the duct system, islet cells, and acini of the human pancreas.^[15,16]^ Recently, a wide variety of tumors, including pancreatic carcinoma, have been shown to secrete ALP into the blood.^[17–20]^ Therefore, the elevation of ALP may indicate a heavy tumor burden. Previously, elevated ALP levels have been shown to correlate with worse survival in hepatocellular carcinoma, gastric carcinoma, neuroendocrine tumors, and metastasis in colorectal cancer.^[21–24]^ Kim et al^[25]^ and Botsis et al^[26]^ found that ALP had prognostic value in pancreatic carcinoma. However, few studies have demonstrated the relationship between LDH and ALP, especially in a prognostic model based on combined LDH and ALP in pancreatic cancer.

Therefore, the aim of this study was to explore the prognostic value of LDH and ALP in patients with pancreatic carcinoma and determine whether the predictive power of LDH combined with ALP was more sensitive than that of either one alone.

## 2 Patients and methods

### 2.1 Ethics statement

Written informed consent was provided to all patients before surgery. The study approval was obtained from independent ethics committees at the First Affiliated Hospital of Sun Yat-sen University. This study was conducted in accordance with the ethical standards of the World Medical Association Declaration of Helsinki.

### 2.2 Study population

A total of 185 patients with resectable pancreatic ductal adenocarcinoma (PDAC) undergoing surgery were recruited in our hospital between July 2005 and December 2010. All patients had pathologically diagnosed PDAC. A routine assessment was performed within 7 days before surgery, including a complete physical examination, hematological and biochemistry profiles, chest X-rays, abdominal ultrasounds, and 3-dimensional contrast-enhanced computed tomography or magnetic resonance imaging.

Our database was interrogated to provide information on all patients who died of PDAC. The eligibility criteria included the tumor–node–metastasis (TNM) stage I, II, III (AJCC, the 7th edition)^[27]^; age 18 to 80 years; and a good performance status (Karnofsky performance scores ≥80). The exclusion criteria were patients with R2 resection (macroscopically positive resection margins); TNM stage IV; existing 2nd malignancy or history of 2nd malignancy in the past 5 years; hematological disorders; perioperative dysfunction of vital organs; and patients who were pregnant at the time of diagnosis. Preoperative serum bilirubin values >2 mg/dL were also excluded to avoid the influence of obstructive jaundice on the ALP value.

### 2.3 Treatment and follow-up

A standard pancreaticoduodenectomy or a pylorus-preserving procedure was performed in patients for ductal carcinoma of the pancreatic head. Patients with PDAC of the body or tail underwent distal pancreatectomy with splenectomy. Right para-aortic lymph node dissection was routinely carried out in pancreatic head tumors, followed by procedures to ensure that no metastatic lymph nodes were found in the frozen section pathology. All patients underwent gemcitabine-based chemotherapy based on the newest edition of the National Comprehensive Cancer Network (NCCN) Guideline. The regimen of gemcitabine alone was 1000 mg/m^2^ (3 times per week followed by a 1 week rest for 6 cycles).

All patients were regularly followed-up through out-patient visits or phone calls according to institutional practice, including ultrasound, X-ray, contrasted computed tomography or magnetic resonance imaging, and tumor markers every 3 to 6 months. Disease-free survival (DFS) was calculated from the date of surgery to the date of recurrence and overall survival (OS) from the date of surgery to the date of PDAC-associated death.

### 2.4 Statistical analysis

Statistical analyses were carried out using SPSS v 20.0 software (Chicago, IL). Student *t* tests were used for the comparison of continuous variables with normal distribution. The Chi-square test was used for categorical variables. The Kaplan–Meier method was used to estimate the survival rates for different groups,^[28,29]^ and the equivalences of the survival curves were tested by log-rank statistics. The Cox proportional hazards model was used for univariate and multivariate survival analyses.^[30]^*P* < 0.05 was considered statistically significant.

## 3 Result

### 3.1 Patients and tumor characteristics

The study contained 81 male patients (43.8%) and 104 female patients (56.2%). The median age was 61 years (range 26–81 years). A total of 161 patients (87.0%) developed recurrence. Tumors were primarily located at the pancreas head (54.6%). The median size of the tumors was 3.0 cm (range 0.9–8.0 cm) in greatest diameter. Ninety-one patients (49.2%) showed poor differentiation, and 90 patients (48.6%) were in stage I based on TNM classification. Increased carbohydrate antigen 19-9 (CA19-9) levels (≥37 U/L) were observed in 138 cases (74.6%), 68 patients (36.7%) had lymph node involvement, and 83 (44.9%) had tumors with perineural invasion detected with histopathological analysis. Microscopic negative resection margin (R0) was achieved in 153 patients (82.7%). Postoperatively, 54 patients (29.2%) developed major complications, while the majority of patients (86.5%) died during follow-up.

### 3.2 Cutoff determination of LDH/ALP

The cutoff values for the enzymes were defined by the upper limit of normal (ULN) values set by the detector (Hitachi 7600 automatic biochemical analyzer) used in our hospital for biochemical analysis. The cutoff values of LDH and ALP were 240 and 110 U/L, respectively. A serum enzyme level above the cutoff value was defined as an increased serum enzyme level.

### 3.3 Correlation between LDH/ALP and clinicopathological characteristics

The clinicopathological characteristics of patients with different LDH/ALP levels were analyzed. An LDH value > 240 U/L was more frequently observed in patients with CA19-9 ≥37 U/L (*P* = 0.003), with lymph node involvement (*P* = 0.001), increased tumor size (*P* = 0.007), greater TNM stage (*P* = 0.04), distant metastasis (*P* = 0.016), and recurrence (*P* = 0.001). Similarly, ALP was positively associated with CA19-9 (*P* = 0.013), lymph node involvement (*P* = 0.029), perineural invasion (*P* = 0.049), tumor size (*P* = 0.004), TNM (*P* = 0.001), distant metastasis (*P* = 0.041), and recurrence (*P* < 0.001) (Table 1). In addition, we found that there was a significant positive correlation between LDH and ALP (*r* = 0.427, *P* < 0.001, Table 2).

**Table 1:**
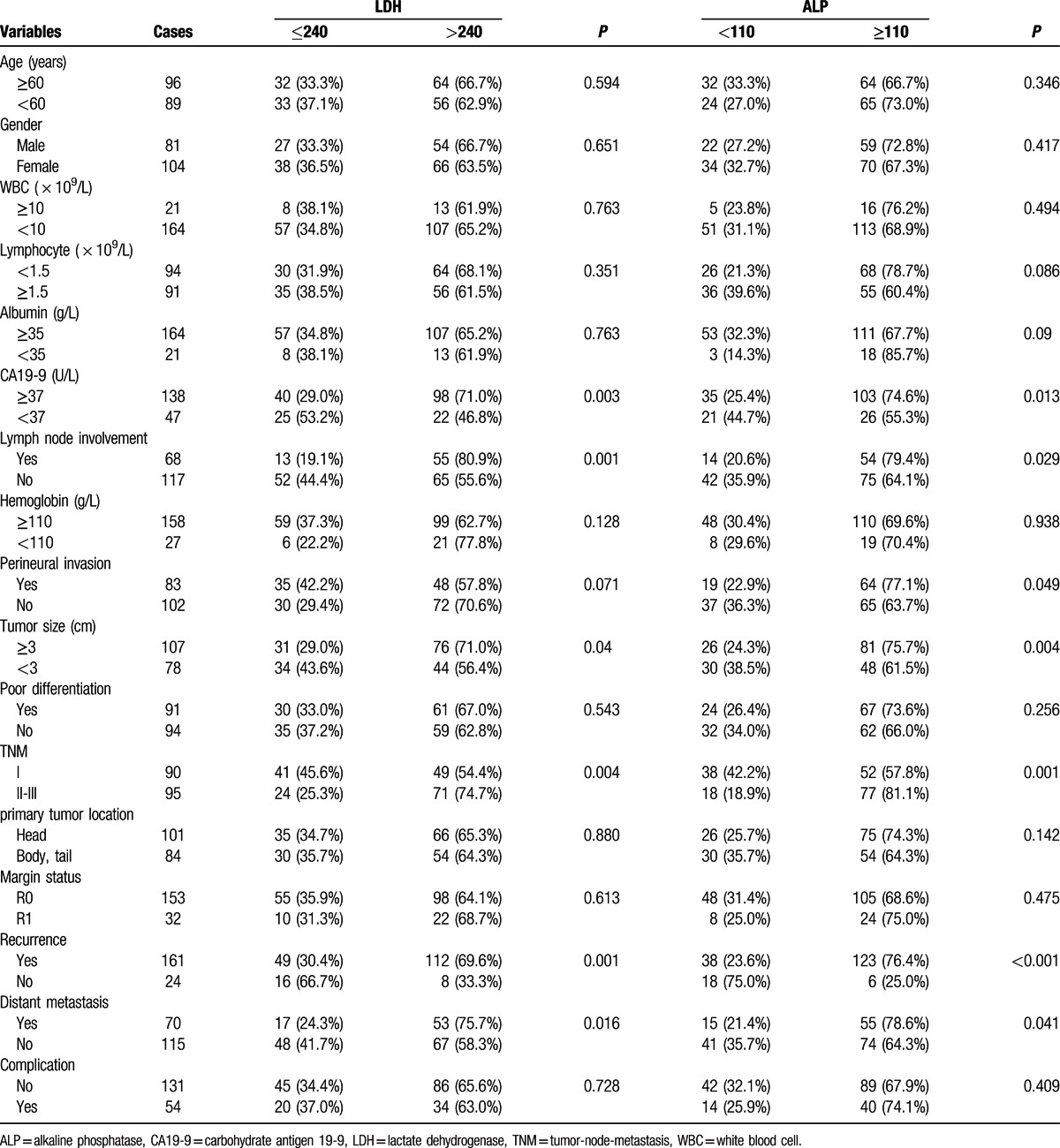
Correlation between the clinicopathological characteristics and LDH/ALP.

**Table 2:**
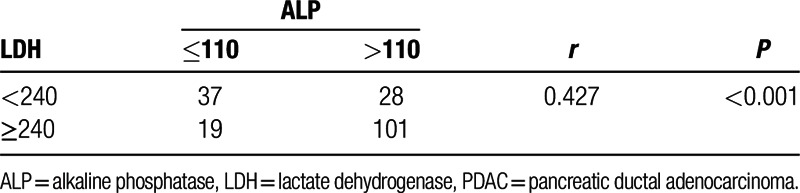
The correlationship between LDH and ALP in PDAC.

### 3.4 Independent prognostic factors of PDAC

To identify the risk factors linked to postoperative DFS and OS, LDH, ALP, and clinicopathological factors were evaluated. The univariate analysis showed that the significant prognostic factors for DFS of PDAC were CA19-9, tumor size, poor differentiation, TNM stage, distant metastasis, LDH, and ALP. Similarly, the significant prognostic factors for OS of PDAC were CA19-9, tumor size, poor differentiation, TNM stage, margin status, distant metastasis, LDH, and ALP (all *P* < 0.05) (Table 3). After multivariate analysis, 4 parameters, including TNM, distant metastasis, LDH, and ALP, were identified as the independent prognostic factors of both DFS and OS (all *P* < 0.05) (Table 4).

**Table 3:**
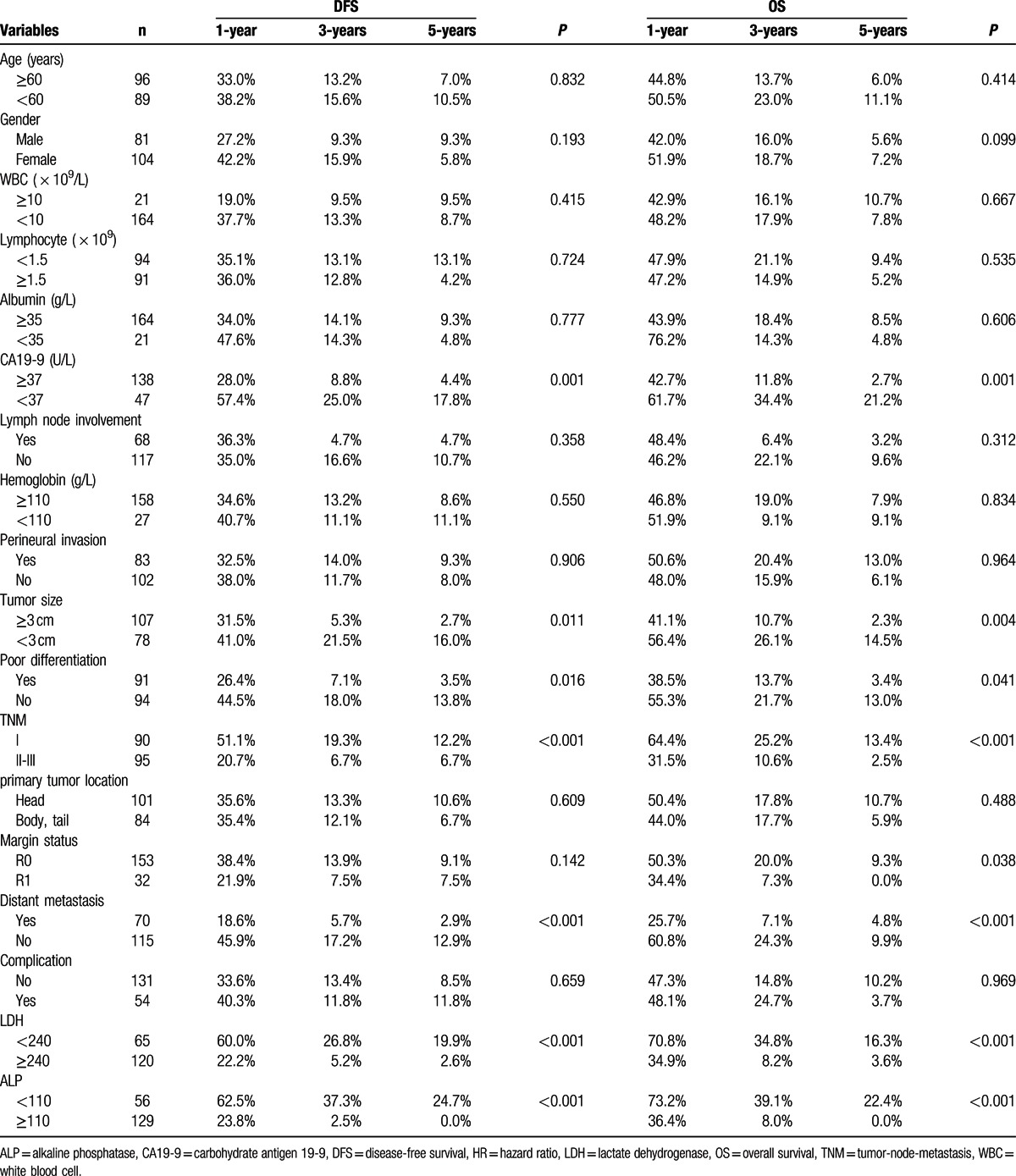
Prognostic factors for DFS and OS by univariate analysis.

**Table 4:**
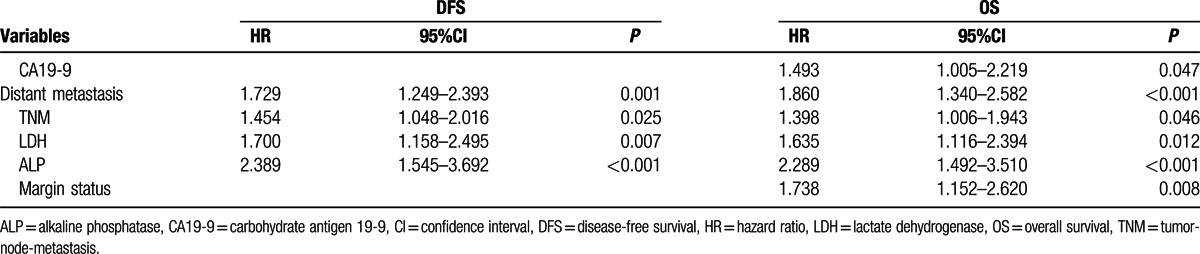
Prognostic factors for disease-free and overall survival by the multivariate Cox proportional hazards regression model.

### 3.5 Elevated LDH and ALP predict PDAC patients’ poor prognosis

**Figure 1:**
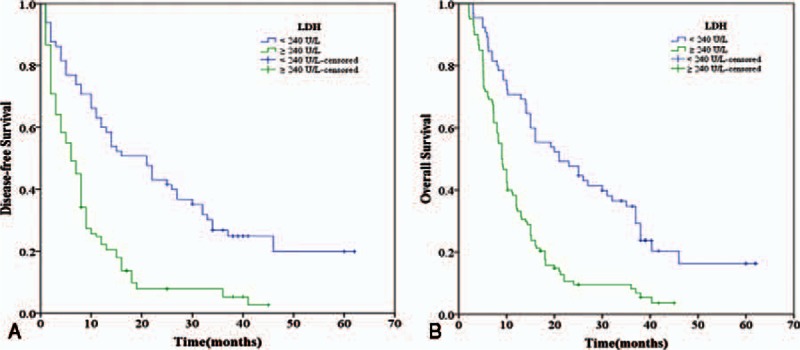
Relationship between LDH and DFS/OS of PDAC patients after surgery. (A) DFS of patients with LDH ≥ 240 (U/L) was significantly shorter than those with LDH < 240 (U/L) (*P* < 0.001, log-rank test). (B) OS of patients with LDH ≥ 240 (U/L) was also markedly shorter than those with LDH < 240 (U/L) (*P* < 0.001, log-rank test). DFS = disease-free survival, LDH = lactate dehydrogenase, OS = overall survival, PDAC = pancreatic ductal adenocarcinoma.

To further test the prognostic value of ALP and LDH in patients with PDAC, we used the Kaplan–Meier method to analyze patients’ survival according to LDH or ALP profiles: LDH < 240 or LDH ≥ 240 (U/L) and ALP < 110 or ALP ≥ 110 (U/L). We found that the 1-, 3-, and 5-year DFS rates of the LDH < 240 U/L group were markedly higher than the LDH ≥ 240 U/L group (60.0%, 26.8%, and 19.9% vs 22.2%, 5.2%, and 2.6%, respectively, *P* < 0.001) (Fig. 1A), while the 1-, 3-, and 5-year OS rates of the LDH < 240 U/L group were also significantly higher than those of the LDH ≥ 240 U/L group (70.8%, 34.8%, and 16.3% vs 34.9%, 8.2%, and 3.6%, respectively, *P* < 0.001) (Fig. 1B). Our findings thus indicated that the elevation of LDH was correlated with poor prognosis of patients with PDAC.

**Figure 2:**
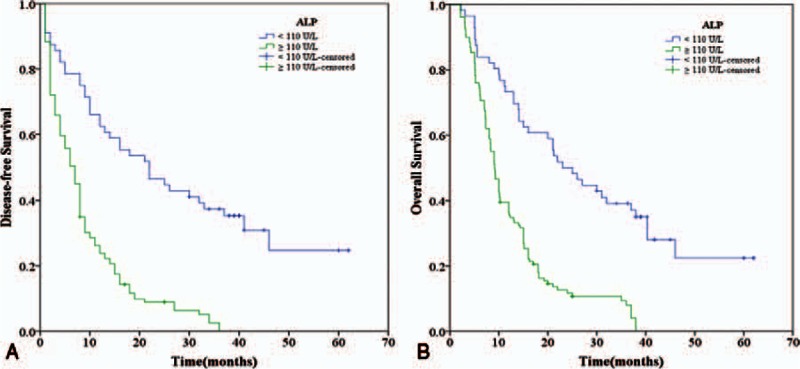
Relationship between ALP and DFS/OS of PDAC patients after surgery. (A) DFS of patients with ALP ≥ 110 (U/L) was significantly shorter than those with ALP < 110 (U/L) (*P* < 0.001, log-rank test). (B) OS of patients with ALP ≥ 110 (U/L) was also significantly shorter than those with ALP < 110 (U/L) (*P* < 0.001, log-rank test). ALP = alkaline phosphatase, DFS = disease-free survival, OS = overall survival, PDAC = pancreatic ductal adenocarcinoma.

Similarly, we found that the 1-, 3-, and 5-year DFS rates of the ALP < 110 U/L group were markedly higher than those of the ALP ≥ 110 U/L group (62.5%, 37.3%, and 24.7% vs 23.8%, 2.5%, and 0.0%, respectively, *P* < 0.001) (Fig. 2A). Additionally, the 1-, 3-, and 5-year OS rates of the ALP < 110 U/L group were significantly higher than those of the ALP ≥ 110 U/L group (73.2%, 39.1%, and 22.4% vs 36.4%, 8.0%, and 0.0%, respectively, *P* < 0.001) (Fig. 2B). Therefore, the elevated ALP was associated poor DFS and OS of PDAC patients as well.

### 3.6 The combination of LDH and ALP shows improved prognostic accuracy for PDAC patients

To analyze the prognostic value of combining LDH and ALP levels for PDAC, we defined each elevation of LDH or ALP as a score of 1, and we divided patients into the following 3 groups: group I, with a score of 0, had a lower level of both ALP (<110 U/L) and LDH (<240 U/L); group II, with a score of 1, had patients with a higher level of ALP (≥110 U/L) and a lower level of LDH (<240 U/L) or patients with a lower level of ALP (ALP < 110 U/L) and a higher level of LDH (≥240 U/L); group III, with a score of 2, consisted of patients with a higher level of both ALP (≥110 U/L) and LDH (≥240 U/L).

**Figure 3:**
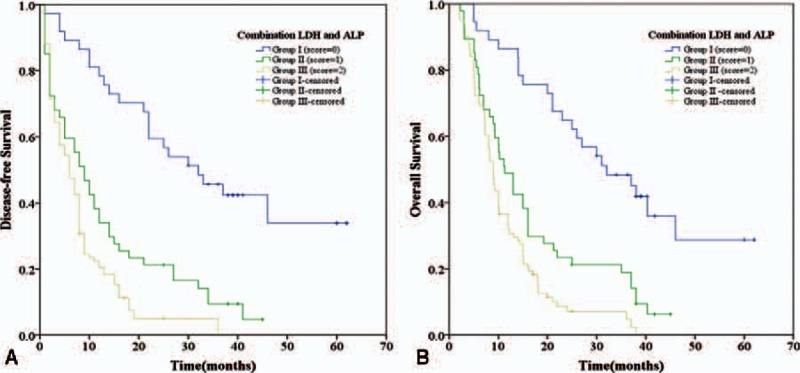
The combination of LDH and ALP was found to enhance the prognostic accuracy for PDAC. Disease-free survival curves (panel A) and overall survival curves (panel B). Group I with low risk, both ALP < 110 (U/L) and LDH < 240 (U/L); group II with middle risk, ALP ≥ 110 (U/L) and LDH < 240 (U/L) or ALP < 110 (U/L) and LDH ≥ 240 (U/L); and group III with high risk, both ALP ≥ 110 (U/L) and LDH ≥ 240 (U/L). ALP = alkaline phosphatase, LDH = lactate dehydrogenase, PDAC = pancreatic ductal adenocarcinoma.

The 1-, 3-, and 5-year DFS rates of group I (score = 0) (78.4%, 45.6%, and 33.9%, respectively) were significantly higher than those of group II (score = 1) (34.0%, 9.5%, and 4.7%, respectively, *P* < 0.001), and group III (score = 2) (20.5%, 0.0%, and 0.0%, respectively, *P* < 0.001) (Fig. 3A). Similarly, the 1-, 3-, and 5-year OS rates of group I (score = 0) (86.5%, 48.4%, and 28.7%, respectively) were also significantly higher than those of group II (score = 1) (48.9%, 18.9%, and 0.0%, respectively, *P* < 0.001) and group III (score = 2) (32.6%, 4.8%, and 0.0%, respectively, *P* < 0.001) (Fig. 3B). Furthermore, we found that the 1-, 3-, and 5-year DFS and OS rates of group II (score = 1) were both significantly higher than those of group III (score = 2) (*P* = 0.016 and *P* = 0.011, respectively) (Fig. 3A and B). These data demonstrate that patients with a score of 0 had the best DFS and OS rates, followed by patients with a score of 1, and patients with a score of 2 had the worst prognosis. Therefore, we defined the patients with a score of 0, 1, and 2 as low, middle, and high risk, respectively.

## 4 Discussion

Tumors consist of cancer cell clones that demonstrate rapid proliferation and invasion. Tumor proliferation and development can cause metabolic changes in some serum enzymes, proteins, and hormones^[22]^; thus, these enzymes, such as LDH and ALP, can reflect tumor progression and may be markers of clinical prognosis. Accumulating evidence^[31]^ has suggested that hypoxia can facilitate cancer development, and Warburg^[32]^ reported that tumor cells may preferentially use the anaerobic pathway of glycolysis in spite of the presence of oxygen. Anaerobic glycolysis may substantially increase the conversion of pyruvate to lactate. LDH, which contains subunit A and B, is a key enzyme of glycolysis during this conversion. A link between LDH-A and c-MYC^[33,34]^ has been verified, and knockdown of LDH-A can significantly diminish tumor growth in a mouse model. Additionally, the relationship among hypoxia, LDH, and tumor-driven angiogenesis has been demonstrated by the excessive activation of hypoxia-inducible factor 1, which regulates gene expression, controls tumor growth, metabolic reprogramming, and aggressiveness.

Another routinely tested serum enzyme in clinical practice is ALP. ALP is a phosphate monoester hydrolase that facilitates the hydrolysis and transfer of phosphate groups in alkaline conditions. Kojima and Sakurada^[35]^ first demonstrated an elevation of ALP activity in mice with Ehrlich ascites tumors. Several other studies^[22,36]^ also showed that ALP was a tumor-associated antigen. It could reflect the proliferation of cancer cells through nucleolar localization. Higher ALP activity in the nucleolus or ALP movement during the cell cycle was linked to tumor proliferation and progression. A hepatocellular carcinoma prognostic index system includes ALP > 200 IU/L as an indicator for poor outcome.^[37]^ Both LDH and ALP are markers of tumor proliferation and progression, and both of them have been confirmed as prognostic markers in hepatocellular carcinoma, esophageal squamous cell carcinoma, nasopharyngeal carcinoma, and pancreatic carcinoma, among others.^[25,31,38,39]^ However, the relationship between LDH and ALP in pancreatic cancer has not been investigated, especially the prognostic value of their combination. We aimed to develop a simple risk assessment model based on LDH and ALP to improve the prediction of recurrence in patients of PDAC, which is an innovation of our research.

In our study, we first analyzed the correlation between LDH/ALP and clinical characteristics and found that an elevated LDH level was positively related to CA19-9 and tumor size. Additionally, patients with increased levels of LDH were more likely to have a higher TNM stage, lymph node involvement, and a higher risk of distant metastasis or recurrence. Similarly, ALP was also positively associated with CA19-9, lymph node involvement, perineural invasion, tumor size, TNM, distant metastasis, and recurrence. All these data indicated that LDH or ALP could not only reflect the tumor burden but also promote tumor progression by influencing tumor metabolism and the microenvironment. Using univariate analysis, we identified many significant prognostic factors for DFS or OS of PDAC, such as CA19-9, tumor size, poor differentiation, TNM stage, distant metastasis, LDH, and ALP. However, after multivariate analysis, we found that the independent predictive factors for both DFS and OS were TNM, distant metastasis, LDH, and ALP.

After further analysis, we found that a shorter postoperative survival of PDAC patients with high levels of LDH had been documented in this study. The 1-, 3-, and 5-year DFS rates and OS rates of patients with high levels of LDH were markedly lower than those of the low level group. As for ALP, the same phenomenon was found. The 1-, 3-, and 5-year DFS rates and OS rates of patients with high levels of ALP were significantly lower than those in the low level group, which was consistent with previous studies.^[25,26]^ As a combination of multiple markers may yield more information for predicting clinical outcome, we combined LDH with ALP to predict the prognosis of PDAC patients. First, we analyzed the correlation between LDH and ALP and found that they were also positively correlated with each other. Then, we separated the patients into 3 groups and developed a simple risk assessment model based on the levels of LDH and ALP. Our results indicated that group I (low level of both ALP and LDH) with a score of 0 had the best prognosis, patients with a score of 1 (higher level of ALP and lower level of LDH or lower level of ALP and higher level of LDH) showed an intermediate prognosis, and patients with a score of 2 (high level of both ALP and LDH) had the worst prognosis. Hence, we defined the patients with a score of 0, 1, and 2 as low, medium, and high risk, respectively. This risk model confirmed our hypothesis that the prognostic accuracy of PDAC can be enhanced through a combination of LDH and ALP.

Several other tumor biomarkers, such as CA19-9, carcinoembryonic antigen (CEA), and CA-50, may also reflect the progression of cancer to some degree. However, their diagnostic sensitivity and accuracy have not been confirmed. CA19-9 has several limitations, such as poor specificity, a lack of expression in the Lewis-negative phenotype, and a higher false-positive rate in the presence of obstructive jaundice.^[40]^ Both CEA and CA-50 have a low sensitivity and specificity, and CEA is the standard tumor marker for screening and predicting the prognosis of colorectal cancer.^[41]^

However, there are still some limitations in the present study. Botsis et al^[23]^ reported that age at diagnosis over 67 years and low levels of albumin were associated with worse prognosis, but there were no associations in our study. These differences may be due to the fact that this is a single-institution, retrospective study. Our results were consistent with the report of Kim et al,^[22]^ who also did not show an association. Thus, a well-designed, prospective study with multicenter involvement and a larger number of patients is needed. In addition, because of the relatively small number of patients, we did not split our dataset into a training dataset and a test dataset for statistical validation, and we hope to validate this in the future.

In conclusion, our study demonstrates that the elevation of preoperative LDH and ALP can be used as prognostic factors for predicting the prognosis of patients with PDAC after surgery. Preoperative LDH and ALP levels provide us with an effective means to identify patients at high risk of recurrence and death. Moreover, their combination can increase the prognostic accuracy for survival of PDAC patients. These findings suggest that treatment plans should consider not only TNM stage but also these prognosis-related serum enzymes. Thus, we can improve individualized therapy for patients with PDAC. However, the exact mechanisms and function of LDH and ALP in PDAC should be elucidated. In the future, this simple preoperative prognostic evaluation could be used to screen patients for personalized therapy.

## Acknowledgments

The authors also thank the guidance of Yi Long education during the writing of this paper.
